# The F score ranks diagnostic tests and prediction models inconsistently with their clinical utility

**DOI:** 10.1186/s41512-025-00214-7

**Published:** 2025-12-08

**Authors:** Melissa Assel, Andrew Vickers

**Affiliations:** Department of Epidemiology and Biostatistics, Memorial Sloan Kettering Cancer Center, New York, NY 10065 USA

**Keywords:** F score, Prediction models, Diagnostic tests, Utility, Net benefit

## Abstract

**Background:**

The F score is derived from precision (positive predictive value) and recall (sensitivity). It is increasingly used to evaluate diagnostic tests and prediction models in the machine learning literature. Although precision and recall can be differentially weighted using a parameter *β*, almost all applications use equal weighting, with *β* set to 1.

**Methods:**

We considered a cancer detection scenario to explore the properties of the F score in comparison to net benefit, a well-established method for evaluating the clinical utility of tests and models. Because missing cancer can be fatal and biopsies are an invasive procedure, we would favor a test with high sensitivity.

**Results:**

F scores did not provide a rank ordering of tests consistent with utility. F1 was highest for a test with greater specificity; in contrast, the conventional decision analytic measure, net benefit, rank ordered tests consistent with clinical intuition, with the highest sensitivity test favored. While it might be argued that F scores can be made consistent with utility by choosing a value of *β* different from 1, we found it is impossible to rationally prespecify *β* for any given clinical scenario, as even small changes in prevalence led to undesirable rank orderings for a given *β* being inconsistent with utility.

**Conclusion:**

The F score ranks diagnostic tests and prediction models inconsistently with their clinical utility. Moreover, the F score does not have an interpretable unit, does not allow for a comparison with a strategy assuming all are negative, and requires dichotomization of models. In contrast, standard decision-analytic measures – net benefit and decision curve analysis – allow rational and consistent choice of weighting, have an interpretable unit, can evaluate the strategy of assuming all are negative, and do not require dichotomization of continuous models. Consistent with TRIPOD AI we recommend that net benefit, alongside discrimination and calibration, be used for the evaluation of diagnostic tests and prediction models.

**Supplementary Information:**

The online version contains supplementary material available at 10.1186/s41512-025-00214-7.

## Introduction

Machine learning approaches to prediction modeling have recently become more common in the medical literature [1]. One interesting corollary has been the reporting of discipline-specific methods of performance evaluation. The F-score, a common metric in machine learning applications outside of medicine [2], was almost unheard of in the medical literature a decade ago. Despite early warnings as to the limitations of the F-score [3], its use has rapidly increased as machine learning is used more widely in medical research: a PubMed search for “F score” or “F1-score” retrieves fewer than 100 citations for 2013 but nearly 3000 in 2023. A similar trend is seen in discipline specific language, with the terms “precision” and “recall” – what are more typically described in medical contexts as positive predictive value and sensitivity – being far more commonly referenced in the contemporary literature. The F score is derived from precision and recall using a weighting factor *β*. As such, we will subsequently refer to this measure as “F*β*”.

F*β* is increasingly being used as a clinical utility measure in the medical literature. For instance, Duan et al. [4] develop several models to predict breast cancer metastases and use F1 as a criterion to select the best model. They draw conclusions in terms of clinical utility, stating that their model has the “potential to be translated into a point-of-care prognostic analysis to reduce breast cancer mortality”. Zhu et al. [5] used F1 to draw a conclusion that they had “established a [heart disease] risk prediction model for patients”.

F*β* has some obvious drawbacks: it does not have an interpretable unit, does not allow for a comparison with a strategy assuming all are negative, and requires dichotomization of models. We were interested in whether, despite these drawbacks, assessing clinical utility using F*β* was a reasonable analytic strategy. Our general approach was to see if we could find obvious scenarios where one of various possible models is clearly preferable and F*β* favored an inferior model, in other words, where using F*β* provides rank ordering that is inconsistent with clinical utility. If this is demonstrated in at least some non-trivial cases, then we can discount any general claim that F*β* is of value. Current guidelines for the evaluation of prediction models [1, 6], whether machine learning or more traditional statistical approaches, recommend the use of net benefit – usually presented as a decision curve analysis – for evaluation of clinical utility. Hence, we also evaluated net benefit in our scenarios.

## Methods

F*β* is calculated from precision (more commonly termed positive predictive value) the proportion of true positives among those who tested positive - and recall (sensitivity) - the proportion of true positives among those who are actual positives. F*β* is the harmonic average of precision and recall weighted by *β*, where *β* is the relative importance of recall to precision. F*β* is defined as [7]:

1 $$\:F\beta\:=\left(1+{\beta\:}^{2}\right)\times\frac{precision\times\:recall}{({\beta\:}^{2}\times\:precision)+recall}$$

where (0 ≤ *β* ≤ ∞). This can be rewritten in terms of the predicted classifiers as:

2 $$\:F\beta\:=\frac{\left(1+{\beta\:}^{2}\right)\times\:True\:Positives}{\left(1+{\beta\:}^{2}\right)\times\:True\:Positives+{\beta\:}^{2}\times\:False\:Negatives+False\:Positives}$$

The *β* parameter of the F*β* can be specified to indicate whether recall or precision should be favored. *β*s greater than 1 imply that recall (sensitivity) is favored and *β*s less than 1 imply precision is favored. However, F1 is by far the most commonly reported in the literature. Indeed, it appears investigators often do not realize that β can be alternatively specified. A PubMed literature search for “F1 score” retrieved 13531 records compared to 88 for “F2 score”, 8 for “F0.5 score” and 2 for “F3 score”. There were 2113 records retrieved by “F score” without specification of β. We reviewed the first 20 records retrieved where Fβ was used to evaluate prediction models or diagnostic tests in human subjects; 9 specified that F1 was used and the other 11 did not specify a specific β, for which it is reasonable to assume that β was set at 1.

Our overall goal was to apply the F score to some illustrative medical research scenarios and determine whether the results of the F score were consistent with our intuitions, for instance, favoring a sensitive over a specific test when it was critical to find all, or nearly all, cases of disease. As a control, we also analyzed the data using net benefit, as this is a standard method recommended in many guidelines including TRIPOD-AI [1].

Net benefit is based on the concept of threshold probability (*pt*). This is the minimum probability of disease at which a decision-maker (a patient or doctor) will opt for treatment. This threshold probability can be expressed in terms of clinical utility as the probability of the event at which the expected utility of treatment is equal to the expected utility of withholding treatment [8, 9]. For example, if a doctor says that they would perform a biopsy if the risk of cancer is 10% (threshold probability) the corresponding odds 10:90 (0.1 (1-0.1.1)=(1/9)) imply that it is nine times worse to delay a cancer diagnosis than to do a biopsy in a patient without cancer. If the model gives the patient a probability of 10% or more, the doctor would do the biopsy but would avoid biopsy if the probability was less than 10%. One can also derive the threshold probability by taking the inverse of the “numbers needed” [10]. For example, a 10% threshold suggests that a clinician would biopsy no more than 10 patients in order to find one cancer.

Threshold probability is used to calculate net benefit using the formula:

3 $$\text{Net benefit} = \frac{\text{True Positives}}{N} - \frac{\text{False Positives}}{N} \times \frac{p_t}{1 - p_t}$$

Net benefit weighs the relative harm of a false positive and a false negative result based on a given threshold probability. The net benefit can be derived from the expected utility of the results of a test (see appendix for a derivation of the net benefit from expected utilities) [11]. Net benefit is the expected utility of treating patients based on the results from a test subtracting the expected utility of treating no patients divided by (or relative to) the benefit of a true positive (compared to a false negative). It is given in the unit of true positives, being the value of a test compared to doing nothing relative to treating a patient with the disease compared to not treating a patient with the disease. For instance, a net benefit of a test of 0.2 means that a test is equivalent to one that identified 20 patients with disease per 100 with no false positives.

Herein we examined the performance of F*β* and net benefit based on various binary classifiers. In our scenario we used an event rate of 20% and we assumed that 4 different binary tests were available (Table 1) with sensitivities and specificities 60% and 90% (“Specific” test), 90% and 60% (“Sensitive” test), 95% and 50% (“Highly Sensitive” test), and 99% and 47% (“Extremely Sensitive” test). We calculated the area under the curve (AUC) as (sensitivity plus specificity) ÷ 2.

**Table 1:** Performance characteristics of the illustrative binary tests for a disease with 20% prevalence

Test	Sensitivity (recall)	Specificity	Positive Predictive Value (precision)	AUC
Assume All Negative	0%	100%	NA	0.50
Assume All Positive	100%	0%	20%	0.50
Specific	60%	90%	60%	0.75
Sensitive	90%	60%	36%	0.75
Highly Sensitive	95%	50%	32%	0.725
Extremely Sensitive	99%	47%	32%	0.73

As our illustrative example, consider we wish to determine if a patient should undergo biopsy for a cancer. The cancer can progress to become incurable if not detected and so we would want a test with high sensitivity. Accordingly, assume most clinicians would decide to biopsy if a patient had anywhere from 5 to 10% risk of cancer, in other words, implying they would biopsy anywhere from 10 to 20 patients in order to identify one cancer. Note that the range of threshold probabilities of interest (5%−10%) depends on the relative harms and benefits of a biopsy, not on the prevalence of a positive biopsy in the population of interest.

We can use that threshold probability to think about the correct the rank ordering of test to detect a cancer with a prevalence of 20%. If the decision to biopsy was determined by the “Highly Sensitive” test (95% sensitivity, 50% specificity) using this test on 10,000 patients would result in biopsying 5900 patients (test positives), finding 1900 cancers (true positives), unnecessarily biopsying 4000 patients (false positives), and missing 100 cancers (false negatives). If the decision to biopsy was determined by the “Sensitive” test (90% sensitivity, 60% specificity) using this test on 10,000 patients would result in biopsying 5000 patients, finding 1800 cancers, performing 3200 unnecessary biopsies, and missing 200 cancers. It is clear that the “Highly Sensitive” test is superior because it is worth doing an extra 900 biopsies to detect 100 additional cancers as 9 biopsies per additional cancer detected is less than 10, the maximum acceptable number of biopsies performed to detect one cancer. Similar math can be used to demonstrate that the ideal ranking is “Extremely Sensitive”, “Highly Sensitive”, “Sensitive”, “Specific”. For instance, the “Extremely Sensitive” test is superior to the “Highly Sensitive” test because it involves biopsying an extra 4 patients per additional cancer found.

We calculated F*β*s for the four tests using different values of *β*. We then altered prevalence to determine the effect on F*β*.

## Results

Table 2 shows the performance characteristics for the binary tests in our illustrative scenario. At a prevalence of 20%, net benefit provides rankings consistent with clinical intuition by placing higher importance on finding the cancer. Net benefit ranks the “Extremely Sensitive” test highest at low threshold probabilities where the harm of a false negative is much worse than a harm of a false positive (Table 2). For thresholds 5% and 10% net benefit ranks the model with 99% sensitivity first followed by the model with 95% then 90% sensitivity as is consistent with clinical intuition. F1 gives a rank ordering in the complete opposite direction to this preference order, with the “Specific” test having the highest F1 and the “Extremely Sensitive” test having the lowest.

**Table 2:** Performance characteristics of the illustrative binary tests at different prevalences

**Prevalence 20%**			
Test	Threshold 5%	Threshold 10%	F1
Assume All Negative	0.0000	0.0000	.%
Assume All Positive	0.1579	0.1111	33%
Specific	0.1158	0.1111	**60%**
Sensitive	0.1632	0.1444	51%
Highly Sensitive	0.1689	0.1456	48%
Extremely Sensitive	**0.1757**	**0.1509**	48%
**Prevalence 12.5%**			
Test	Threshold 5%	Threshold 10%	F1
Assume All Negative	0.0000	0.0000	.%
Assume All Positive	0.0789	0.0278	22%
Specific	0.0704	0.0653	**52%**
Sensitive	0.0941	**0.0736**	38%
Highly Sensitive	0.0957	0.0701	35%
Extremely Sensitive	**0.0993**	0.0722	35%
**Prevalence 9%**			
Test	Threshold 5%	Threshold 10%	F1
Assume All Negative	0.0000	0.0000	.%
Assume All Positive	0.0421	−0.0111	17%
Specific	0.0492	**0.0439**	**46%**
Sensitive	0.0618	0.0406	30%
Highly Sensitive	0.0616	0.0349	27%
Extremely Sensitive	**0.0637**	0.0355	27%

Net benefit at 5% and 10% threshold probabilities and F with a β value of 1 are displayed. The highest values for each performance metric are bolded indicating the optimal model based on the respective performance metric

Table 2 also shows the results if the prevalence of disease is changed. Even small changes in prevalence affect the rank ordering of tests based on net benefit at a given threshold and the F score at a given *β*. Critically however, these changes in rank ordering based on net benefit are congruent with clinical utility whereas those based on the F score are not. F1 ranks the “Specific” test highest across all prevalences and it is only consistent with net benefit in the situation where the prevalence is lower than the threshold probability (10% threshold probability at a 9% prevalence) where it would be unusual to use a diagnostic test or prediction model to inform decision-making. Table 2 shows that at a threshold probability of 10%, the “Extremely Sensitive” test has the highest net benefit if prevalence is 20%, but if prevalence is 12.5% or 9%, the “Sensitive” test is preferred. Supplemental Table 2 illustrates the clinical consequences of using these tests to determine biopsy at various prevalences on 10,000 patients. Consider we would want to do no more than 10 additional biopsies per additional cancer detected. At the corresponding 10% threshold probability, the “Extremely Sensitive” test has higher utility than the “Specific” test as doing an extra 1220 biopsies to detect 180 additional cancers translates to an additional 7 biopsies performed per additional cancer detected, which is less than our threshold of 10. Conversely at the 12.5% and 9% prevalences the number of additional biopsies needed to detect an additional cancer is greater than 10 when using the “Extremely Sensitive” test compared to the “Sensitive” test indicating the “Sensitive” has higher utility. These rankings are consistent with net benefit at the 10% threshold probability. Supplemental Fig. 1 illustrates Fβ and net benefit by prevalence for a given β and threshold probability; net benefit is a linear function of prevalence while Fβ is not. Additionally, a fixed β cannot provide rank orderings consistent with net benefit as these two functions are not monotonic transformations of each other.

It might be argued that the poor performance of the F score in Table 2 was due to an inappropriate choice of *β*. Such a view might accept that researchers almost always set *β* to 1 but point out that this is not an inherent issue with the F score, but how it is used in practice: more appropriate choice of *β* would result in better properties of the F score. Supplemental Table 1 shows the rank ordering of tests for various β values. From our literature review, we found that F2 was most commonly used in scenarios where sensitivity would be favored. However, F2 also fails to rank order correctly: at the 20% threshold F2 favors the “Extremely Sensitive” test but then ranks the “Sensitive” higher than the “Highly Sensitive” test. F3 does rank order correctly, but if we wish to use a 10% threshold probability, at prevalence 12.5%, the rank ordering of F3 is no longer consistent with net benefit, favoring the “Extremely Sensitive” rather than the “Sensitive” test. To see that net benefit is giving the intuitively correct answer, the optimal strategy based on a 10% threshold (“Sensitive test”; 90% sensitivity 60% specificity), would for every 10,000 patients, biopsy 4625 patients and find 1125 cancers; the “Extremely Sensitive” test would biopsy 5875 patients to find 1238 cancers, meaning that 1250 extra patients would be biopsied to find any additional 113 cancers (Supplemental Table 2). This translates to an additional 11 biopsies per cancer detected which is more than our clinically acceptable ratio of 10 additional biopsies per cancer, accordingly we would opt not to perform these extra biopsies indicated with the “Extremely Sensitive” test and instead use the “Sensitive” test. Consider, alternatively we wish to use a 5% threshold probability, at prevalences of 20% and 12.5%, F3 ranks consistently with the 5% threshold. However, Supplemental Table 1 shows that at a prevalence of 9%, the highest net benefit at a threshold of 5% is for the “Extremely Sensitive” test, but the highest F3 value is consistent with the “Sensitive” test.

**Fig. 1:**
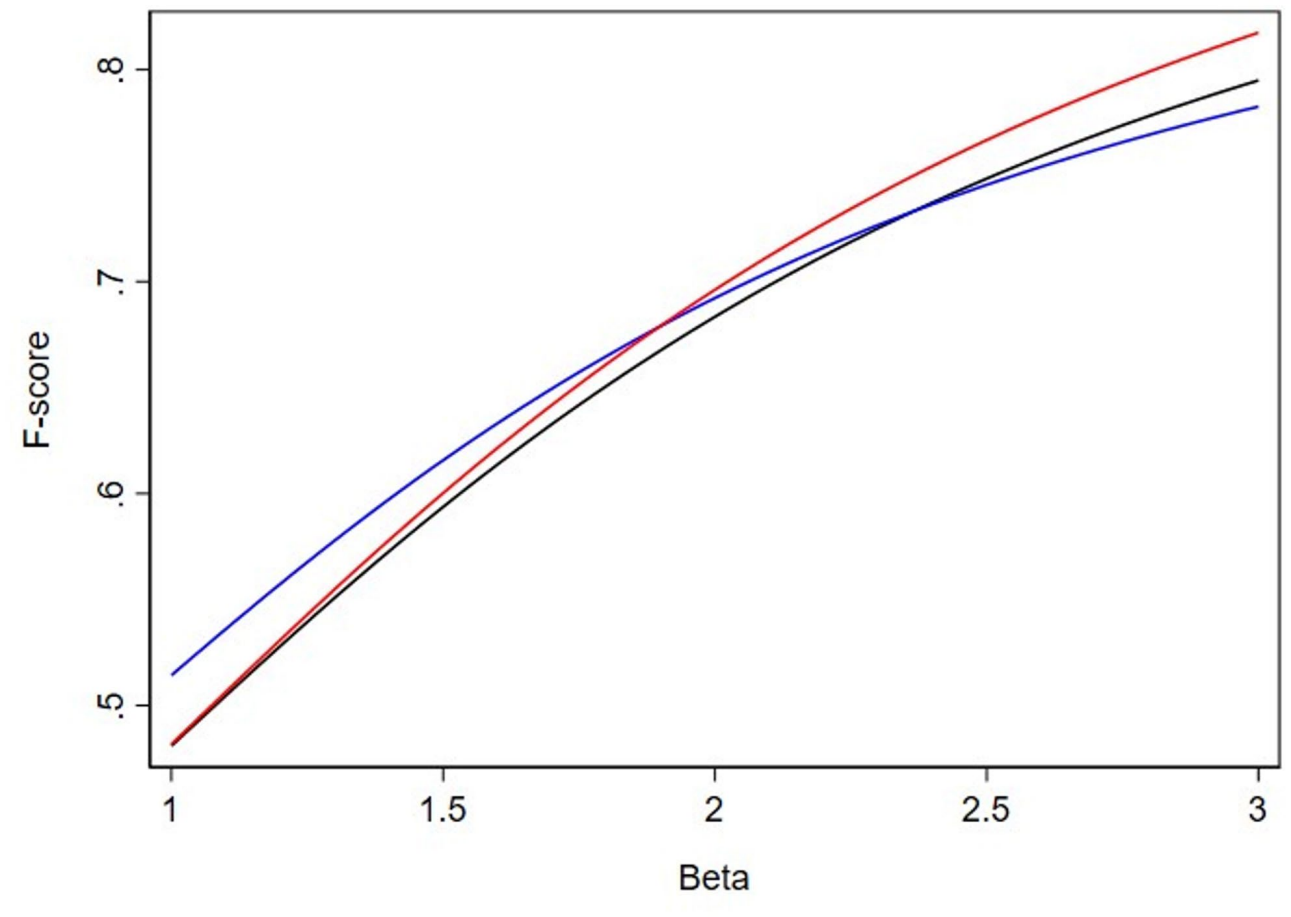
F*β *scores at prevalence 20%. The red line represents the test with 99% sensitivity and 47% specificity (“Extremely Sensitive”), the black line represents a test with 95% sensitivity and 50% specificity (“Highly Sensitive”), and the blue line represents the test with 90% sensitivity and 60% specificity (“Sensitive”)

**Fig. 2:**
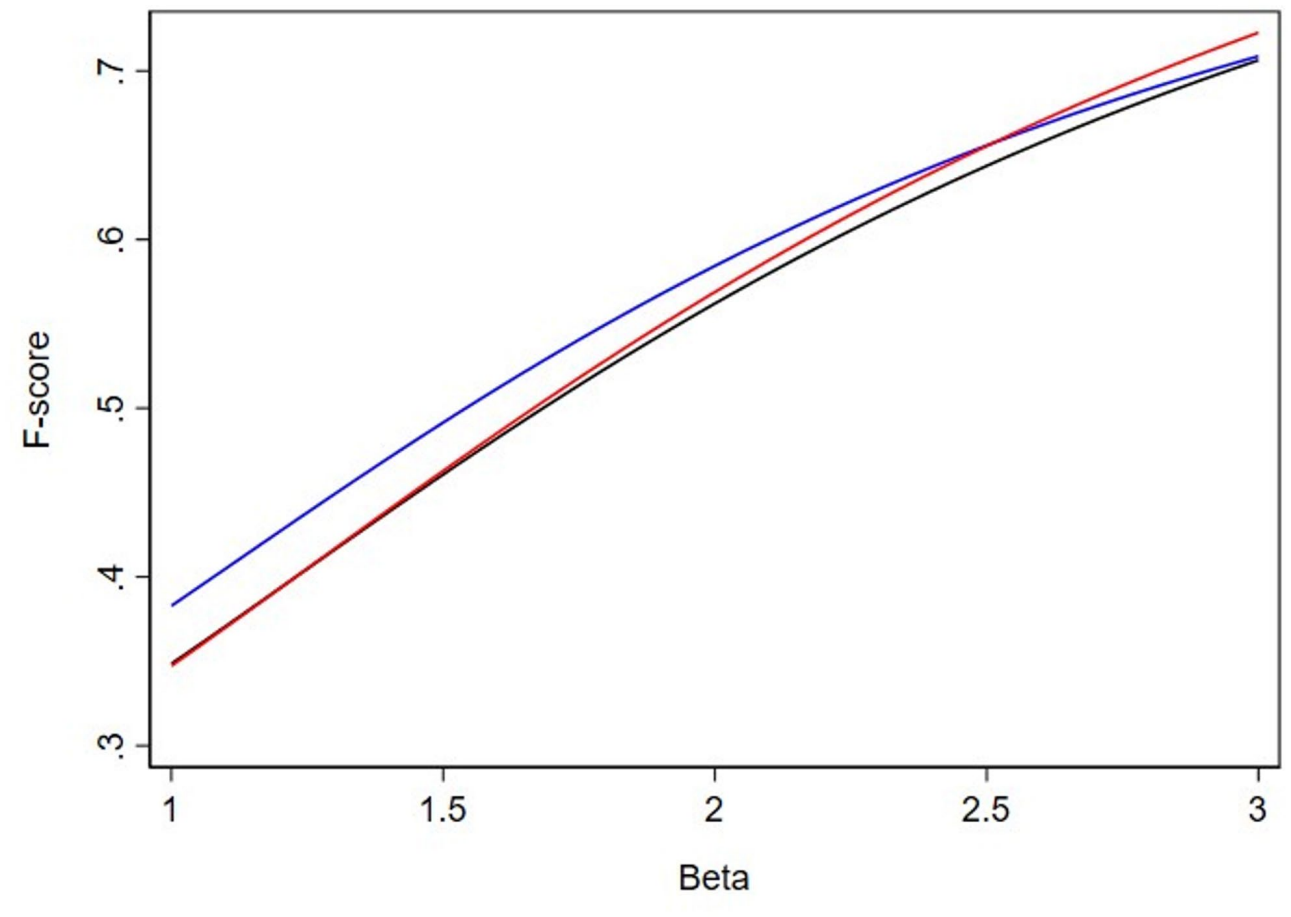
F*β* scores at prevalence 12.5%. The red line represents the test with 99% sensitivity and 47% specificity (“Extremely Sensitive”), the black line represents a test with 95% sensitivity and 50% specificity (“Highly Sensitive”), and the blue line represents the test with 90% sensitivity and 60% specificity (“Sensitive”)

**Fig. 3:**
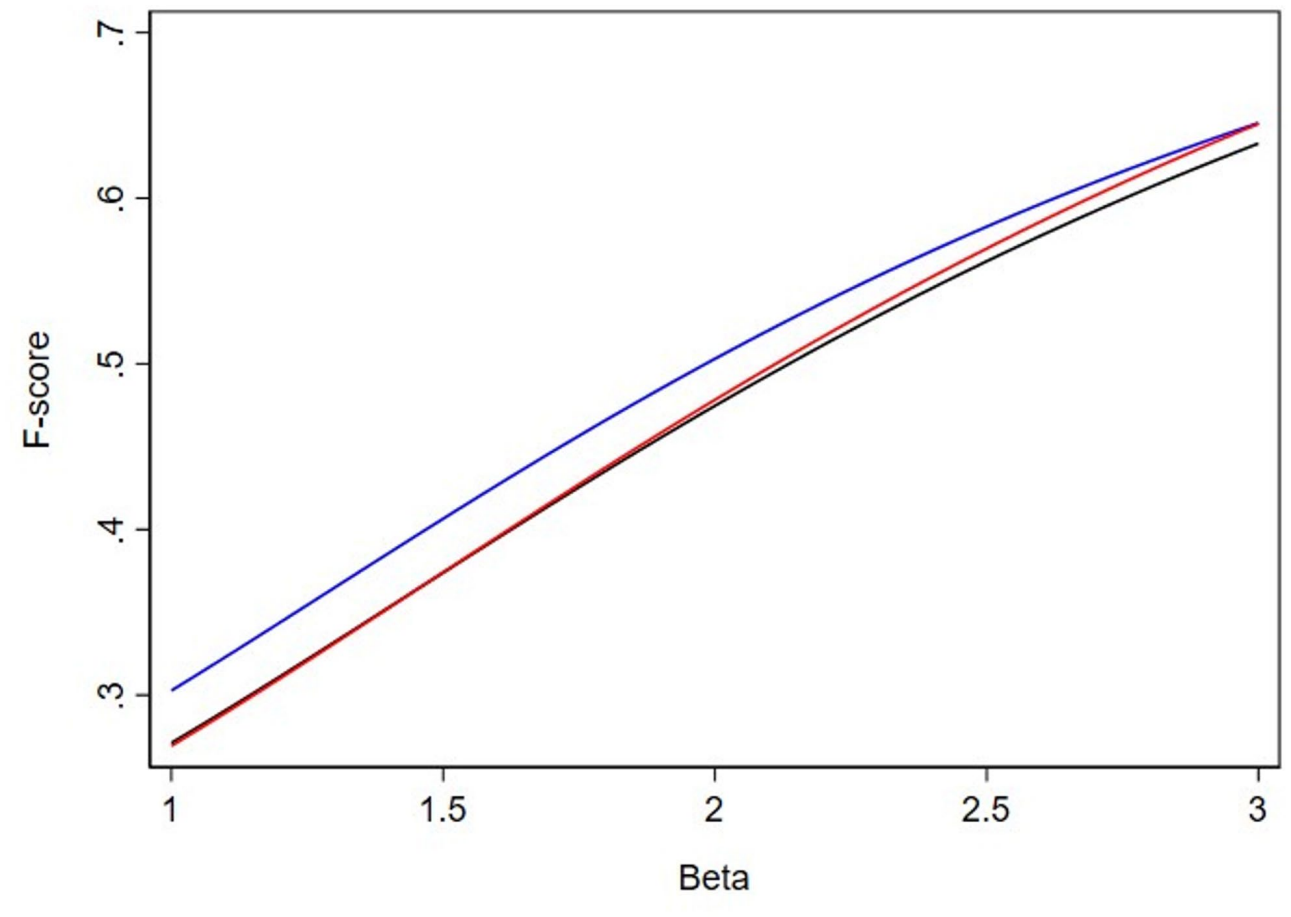
F*β* scores at prevalence 9%. The red line represents the test with 99% sensitivity and 47% specificity (“Extremely Sensitive”), the black line represents a test with 95% sensitivity and 50% specificity (“Highly Sensitive”), and the blue line represents the test with 90% sensitivity and 60% specificity (“Sensitive”)

To further explore how slight modifications to the relative weighing of recall to precision results in different tests being selected by F*β*, we generated Figs. 1, 2 and 3. This displays the relationship of F*β*s between 3 binary tests for a disease with 20%, 12.5% and 9% prevalence across a range of *β* values. Looking at Fig. 1, clinical intuition tells us that we should select the test with the highest sensitivity (99%, red line), but it is not until we reach a *β* value of greater than 1.9 that the F*β* does indeed rank highest the test with the highest sensitivity. Consider that, if the 99% “Extremely Sensitive” test was not available, we should select the test with 95% sensitivity (black line) when compared to the test with 90% sensitivity (blue line) but it is not until we reach a *β* value of greater than 2.4 that F*β* ranks the test with 95% sensitivity (black line) higher than the test with 90% sensitivity (blue line). Certainly, it is difficult to conceptualize differences of *β* values of 2.3 versus 2.5 in terms of the weighing of precision to recall. However, we demonstrate that this level of fine tuning of the *β* parameter is necessary to provide rank orderings of the F*β* consistent with clinical intuition [4].

In summary, F*β* does not generally provide rank orderings consistent with intuition, such as selecting the test with the highest sensitivity to find an aggressive cancer. Proponents of F*β* do acknowledge choice of *β* is critical to achieve the correct rank ordering of tests but here we demonstrate that even subtle changes in prevalence result in rank orderings inconsistent with utility for a given *β*.

## Discussion

We demonstrate that the F1 score, by far the most widely used F score, gives rank ordering of tests and models that are inconsistent with clinical intuition, for instance, it fails to favor a sensitive test in a cancer detection scenario. In contrast, net benefit, a widely recommended measure of clinical utility, always gave an appropriate rank ordering consistent with clinical intuition. We also demonstrate that while in principle, an analyst can vary *β* in an attempt to better reflect a clinical situation, incorrect specification of the *β* parameter will give an inappropriate rank ordering of tests. Moreover, we demonstrate even small changes in prevalence can alter the optimal *β* parameter. As such, it is difficult to imagine a setting where the population prevalence for which the test or model would be applied is known precisely and the analyst can then specify a relationship between precision and recall that would give a result congruent with net benefit. Thus, it is impossible to rationally prespecify β for any given clinical scenario. Moreover, we would further argue that the weighting of precision to recall is not intuitive and is thus challenging for an investigator to specify. It is certainly difficult to conceptualize the difference between a scenario where recall is deemed twice as important as precision to a scenario where recall is deemed three times as important as precision. We have not seen a clear worked example of how a decision-maker could rationally choose a specific weighting between precision and recall.

This contrasts with net benefit, where the parameter the analyst must define is threshold probability, the minimum probability of event at which treatment would be pursued. Patients and doctors will generally have a reasonable answer for questions such as “what is the probability of finding a cancer for which you would perform a biopsy?” or “at what probability of having positive lymph nodes would you perform a lymph node dissection?”. Indeed, such answers are necessary for clinical implementation of any prediction model. If a doctor is unable to answer the question about the comparative harms of biopsy and diagnostic delay, they will be unable to use a cancer prediction model because the results of the prediction model – the patient’s risk of cancer - will be uninterpretable.

We see three further disadvantages of the F*β*, as mentioned in the introduction. The first concerns the unit. The units of F*β* do not lend themselves to interpretations useful for assessing clinical utility despite what has been claimed in some recent publications in the medical literature. It is unclear what a F2 score of 69% means in terms of patients and outcomes. While there have been proposals for monotonic transformations of the F*β* that are claimed to provide a more clinically interpretable unit, we question whether this is in fact the case [12]. For instance, the F* metric has been defined as: “the number of correctly classified class 1 objects expressed as a fraction of the number of objects which are not correctly classified class 0 objects.” In contrast, the unit of net benefit is true positives [10]. Net benefit incorporates the odds at the threshold probability which reflects how one weighs the relative harm of a false-positive relative to the benefit of a true positive [13]. For instance, the net benefit of 0.1509 at a threshold of 10% means that using the “Extremely Sensitive” test to determine which patient gets biopsied is the equivalent of a strategy that would result in biopsying 1509 patients per 10,000, all of whom had cancer. Net benefit can also be expressed in terms of a “net reduction in interventions”, for instance, we might calculate that using the “Extremely Sensitive” test is the equivalent of a test that found the same number of cancers if all patients were biopsied, but with 3586 fewer biopsies per 10,000.

The second disadvantage of F*β* is that it requires the dichotomization of output. This is not optimal for medical settings because there are differences between patients both in terms of co-morbidities and personal preferences that affect the choice of threshold. For instance, a younger, healthy patient worried about cancer would have a lower threshold for biopsy than an older patient with comorbidities and an aversion to invasive procedures. This is why medical prediction models generally produce an output in percentage risk from 1 to 99% and why discrimination and calibration are calculated without dichotomization. This is also why the standard method to assess clinical consequences of implementing a prediction model, decision curve analysis, evaluates net benefit across a range of reasonable threshold probabilities [9].

The third disadvantage of F*β* scores is that they are not calculable for the strategy of assuming all patients are negative, even though this may be the most desirable strategy. Take the case of a cancer with prevalence of 0.1% where a doctor has told us that, given the harms and risks of biopsy, they would perform no more than 20 biopsies in order to find one cancer, a threshold for biopsy of 5%. Using an available test with 80% sensitivity and 90% specificity on 1000 patients would lead to 206 patients being biopsied, with 8 cancers found. The positive predictive value of the test is < 0.5%, so even were a patient to test positive, their risk is far below the threshold required for biopsy. Accordingly, the optimal strategy is to ignore the test and biopsy no-one, and indeed, this is the strategy with the highest net benefit. This is far from an unusual case: there are many cancers, where a test is available but is not used in practice. Analysis by F*β* would not be able to determine that the test should not be used and instead, no patient should be biopsied.

## Conclusion

F*β*, or the F score, ranks diagnostic tests and prediction models inconsistently with their clinical utility. Moreover, F*β* does not have an interpretable unit, does not allow for a comparison with a strategy assuming all are negative, and requires dichotomization of models. In contrast, the standard recommended method for assessing clinical utility in guidelines such as TRIPOD AI [1] - decision curve analysis using net benefit - allows rational and consistent choice of weighting, has an interpretable unit, can evaluate the strategy of assuming all are negative and does not require dichotomization of continuous models. We recommend that F*β* be avoided and that net benefit, alongside discrimination and calibration, be used instead for model evaluation.

## Supplementary Information

Supplementary Material and Appendix

## Data Availability

Not Applicable.

## References

[1] Collins GS, Moons KGM, Dhiman P, Riley RD, Beam AL, Van Calster B, et al. Tripod + AI statement: updated guidance for reporting clinical prediction models that use regression or machine learning methods. BMJ. 2024;385:078378. 10.1136/bmj-2023-078378. 10.1136/bmj-2023-078378 PMC11019967 38626948

[2] Sokolova M, Japkowicz N, Szpakowicz S. Beyond accuracy, f-score and roc: A family of discriminant measures for performance evaluation. In: Sattar, A., Kang, B.-h, editors AI 2006: Advances in Artificial Intelligence. Berlin, Heidelberg, ??? (2006).

[3] Sokolova M, Lapalme G. A systematic analysis of performance measures for classification tasks. Inf Process Manag. 2009;45(4):427–37.

[4] Duan H, Zhang Y, Qiu H, Fu X, Liu C, Zang X, et al. Machine learning-based prediction model for distant metastasis of breast cancer. Comput Biol Med. 2024;169:107943. 10.1016/j.compbiomed.2024.107943. 38211382 10.1016/j.compbiomed.2024.107943

[5] Zhu H, Qiao S, Zhao D, Wang K, Wang B, Niu Y, et al. Machine learning model for cardiovascular disease prediction in patients with chronic kidney disease. Front Endocrinol (Lausanne). 2024;15:1390729. 10.3389/fendo.2024.1390729. 38863928 10.3389/fendo.2024.1390729 PMC11165240

[6] Efthimiou O, Seo M, Chalkou K, Debray T, Egger M, Salanti G. Developing clinical prediction models: a step-by-step guide. BMJ. 2024;386:078276. 10.1136/bmj-2023-078276. 10.1136/bmj-2023-078276 PMC11369751 39227063

[7] Chinchor N. MUC-4 evaluation metrics. In: Fourth Message Understanding Conference (MUC-4): Proceedings of a Conference Held in McLean, Virginia, June 16–18, 1992 (1992). https://aclanthology.org/M92-1002/

[8] Pauker SG, Kassirer JP. The threshold approach to clinical decision making. N Engl J Med. 1980;302(20):1109–17. 10.1056/NEJM198005153022003. 7366635 10.1056/NEJM198005153022003

[9] Vickers AJ, Elkin EB. Decision curve analysis: a novel method for evaluating prediction models. Med Decis Mak. 2006;26(6):565–74. 10.1177/0272989x06295361. 10.1177/0272989X06295361 PMC2577036 17099194

[10] Vickers AJ, Van Calster B, Steyerberg EW. Net benefit approaches to the evaluation of prediction models, molecular markers, and diagnostic tests. BMJ. 2016;352:6. 10.1136/bmj.i6. 10.1136/bmj.i6 PMC4724785 26810254

[11] Vickers AJ, Cronin AM, Gonen M. A simple decision analytic solution to the comparison of two binary diagnostic tests. Stat Med. 2013;32(11):1865–76. 10.1002/sim.5601. 22975863 10.1002/sim.5601 PMC3531575

[12] Hand DJ, Christen P, Kirielle N. F*: an interpretable transformation of the f-measure. Mach Learn. 2021;110(3):451–6. 10.1007/s10994-021-05964-1. 33746357 10.1007/s10994-021-05964-1 PMC7958589

[13] Vickers AJ, Calster B, Steyerberg EW. A simple, step-by-step guide to interpreting decision curve analysis. Diagn Prognostic Res 3(1), 18. 10.1186/s41512-019-0064-7 PMC6777022 31592444

