# Supplementary material for: The F score ranks diagnostic tests and prediction models inconsistently with their clinical utility
Source: Diagn Progn Res. 2025 Dec 8;9:30. doi: 10.1186/s41512-025-00214-7 (PMC12683848; doi:10.1186/s41512-025-00214-7)
Supplement: Supplementary file 1 — Supplementary Material and Appendix [file 41512_2025_214_MOESM1_ESM.pdf]

Supplementary Tables

Supplemental Table 1 Performance characteristics of the illustrative binary tests at different prevalences giving additional F scores

**Prevalence 20%**					
Test	Threshold 5%	Threshold 10%	F1	F2	F3
Assume All Negative	0.0000	0.0000	.%	.%	.%
Assume All Positive	0.1579	0.1111	33%	56%	71%
Specific	0.1158	0.1111	**60%**	60%	60%
Sensitive	0.1632	0.1444	51%	69%	78%
Highly Sensitive	0.1689	0.1456	48%	68%	79%
Extremely Sensitive	**0.1757**	**0.1509**	48%	**70%**	**82%**
**Prevalence 12.5%**					
Test	Threshold 5%	Threshold 10%	F1	F2	F3
Assume All Negative	0.0000	0.0000	.%	.%	.%
Assume All Positive	0.0789	0.0278	22%	42%	59%
Specific	0.0704	0.0653	**52%**	57%	58%
Sensitive	0.0941	**0.0736**	38%	**58%**	71%
Highly Sensitive	0.0957	0.0701	35%	56%	71%
Extremely Sensitive	**0.0993**	0.0722	35%	57%	**72%**
**Prevalence 9%**					
Test	Threshold 5%	Threshold 10%	F1	F2	F3
Assume All Negative	0.0000	0.0000	.%	.%	.%
Assume All Positive	0.0421	-0.0111	17%	33%	50%
Specific	0.0492	**0.0439**	**46%**	**53%**	57%
Sensitive	0.0618	0.0406	30%	50%	**65%**
Highly Sensitive	0.0616	0.0349	27%	47%	63%
Extremely Sensitive	**0.0637**	0.0355	27%	48%	64%

Net benefit at a 5% and 10% threshold probabilities and F with β values of 1, 2, and 3 are displayed. The highest values for each performance metric are bolded indicating the optimal model based on the respective performance metric.

Supplemental Table 2 Clinical consequences of tests by prevalence for 10,000 patients

**Prevalence 20%**				
Test	Number Biopsied (Test Positives)	Unnecessary Biopsies (False Positives)	Cancers Detected (True Positives)	Cancers Missed (False Negatives)
Sensitive	5000	3200	1800	200
Extremely Sensitive	6220	4240	1980	20
Difference	1220	1040	180	180
Biopsies per additional cancer 1220/180=7				
**Prevalence 12.5%**				
Test	Number Biopsied (Test Positives)	Unnecessary Biopsies (False Positives)	Cancers Detected (True Positives)	Cancers Missed (False Negatives)
Sensitive	4625	3500	1125	125
Extremely Sensitive	5875	4627	1238	12
Difference	1250	1137	113	113
Biopsies per additional cancer 1250/113=12				
**Prevalence 9%**				
Test	Number Biopsied (Test Positives)	Unnecessary Biopsies (False Positives)	Cancers Detected (True Positives)	Cancers Missed (False Negatives)
Sensitive	4450	3640	810	90
Extremely Sensitive	5714	4823	891	9
Difference	1264	1183	81	81
Biopsies per additional cancer 1264/81=16				

The number of biopsies per additional cancer detected is calculated as the number of additional biopsies performed and cancers detected using the "Extremely Sensitive" test versus the "Sensitive" test and rounded up to the nearest whole number. For a threshold of 10%, net benefit is highest for the "Extremely Sensitive" test at the prevalence of 20% and for the "Sensitive" test at prevalence of 12.5% and 9%. This is congruent with clinical utility as a 10% threshold implies it would be worth doing no more than 10 biopsies to find one cancer. The number of additional biopsies for additional cancer found when moving from the "Sensitive" to "Extremely Sensitive" test is less than 10 only if prevalence is 20%.

Supplementary Figure

Supplemental Figure 1 F1 and net benefit at a 10% threshold probability by prevalence)

The red line represents the test with 99% sensitivity and 47% specificity ("Extremely Sensitive"), the black line represents a test with 95% sensitivity and 50% specificity ("Highly Sensitive"), and the blue line represents the test with 90% sensitivity and 60% specificity ("Sensitive").

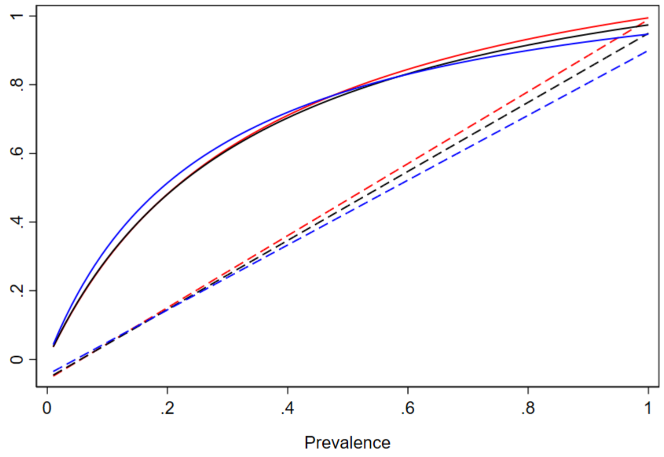

Appendix A Derivation of Net Benefit

Here we correct an algebraic error in a prior publication [**?**] for the derivation of net benefit. **?**] assert that when the probability of disease is identical to the threshold probability the expected utilities of treatment and no treatment are equal. When these equalities are rearranged to be expressed in terms of the odds at the threshold probability, we see that this quantity equals the benefit of a true negative (compared to a false positive) relative to, or divided by, the benefit of a true positive (compared to a false negative). In other words, the odds at the threshold probability is the benefit of forgoing treatment among those without the disease divided by the benefit of treatment among those with the disease. Where u_{xy} is the utility to the patient of the outcome where x is the treatment and y is the true disease state and p_t is the threshold probability.

$$\frac{u_{00} - u_{10}}{u_{11} - u_{01}} = \frac{p_t}{1 - p_t}$$

Using T to indicate the result of a diagnostic test and D for true disease status the utility associated with the four possible outcomes of a diagnostic test are as follows:

$$\text{True Positive} = P(T = 1|D = 1) \cdot P(D = 1) \cdot u_{11}$$
$$\text{False Negative} = P(T = 0|D = 1) \cdot P(D = 1) \cdot u_{01}$$
$$\text{False Positive} = P(T = 1|D = 0) \cdot P(D = 0) \cdot u_{10}$$
$$\text{True Negative} = P(T = 0|D = 0) \cdot P(D = 0) \cdot u_{00}$$

The expected utility of using a test (u_{test}) with trivial costs, harms, or inconvenience is the sum of the utilities across all possible outcomes:

$$u_{\text{test}} = P(T = 1|D = 1) \cdot P(D = 1) \cdot u_{11} + P(T = 0|D = 1) \cdot P(D = 1) \cdot u_{01}$$

$$+P(T = 1|D = 0) \cdot P(D = 0) \cdot u_{10} + P(T = 0|D = 0) \cdot P(D = 0) \cdot u_{00}$$

The utility of treating no patients is:

$$u_{\text{none}} = P(D = 1) \cdot u_{01} + P(D = 0) \cdot u_{00}$$

Expressing this equality in terms of the complements of the true negative and false negative utilities we can express the improvement in utility of using a test compared to treating no one as:

$$u_{\text{test}} - u_{\text{none}} = P(T = 1|D = 1) \cdot P(D = 1) \cdot (u_{11} - u_{01}) +$$
$$P(T = 1|D = 0) \cdot P(D = 0) \cdot (u_{10} - u_{00})$$

$$u_{\text{test}} - u_{\text{none}} = \frac{P(T = 1 \cap D = 1)}{P(D = 1)} \cdot P(D = 1) \cdot (u_{11} - u_{01}) +$$
$$\frac{P(T = 1 \cap D = 0)}{P(D = 0)} \cdot P(D = 0) \cdot (u_{10} - u_{00})$$

$$u_{\text{test}} - u_{\text{none}} = \frac{\text{True Positive Count}}{n} \cdot (u_{11} - u_{01}) - \frac{\text{False Positive Count}}{n} \cdot (u_{00} - u_{10})$$

Where the utility of a test relative to treating no one is the utility of treatment among those with the disease multiplied by the proportion of true positives minus the utility of no treatment among those without the disease multiplied by the proportion of false positives.

Which can be rewritten as:

$$\frac{u_{\text{test}} - u_{\text{none}}}{u_{11} - u_{01}} = \frac{\text{True Positive Count}}{n} - \frac{\text{False Positive Count}}{n} \cdot \frac{p_t}{1 - p_t}$$

Where the above equation is net benefit. Expressed as utilities, the net benefit is the expected increase in utility associated with treating patients based on a test compared to treating no patients relative the increase in utility of treating a patient with the disease compared to not treating a patient with the disease. Thus, the units of net benefit are relative to the utility of a true positive treated.
